# Ultrabioconformal, Self-Healable, and Antioxidized Polydopamine-Inspired Nanowire Hydrogels Enable Resolving Power in Forehead and Ear Electroencephalograms for Brain Function Assessment

**DOI:** 10.1021/acsami.4c23013

**Published:** 2025-04-17

**Authors:** Kanishk Singh, Chun-Chang Lin, Wei-Han Huang, Wan-Lou Lei, Herming Chiueh, Yu-Han Wang, Po-Hsueh Chang, Ru-Zheng Lin, Wei-Chen Huang

**Affiliations:** 1 Department of Electronics and Electrical Engineering, National Yang Ming Chiao Tung University1001 University Rd., Hsinchu City 30010, Taiwan; 2 The Affiliated Senior High School of National Taiwan Normal University, No. 143, Sec. 3, Xinyi Rd., Taipei City 106348, Taiwan; 3 Low Carbon and Energy Storage Division, Green Energy & Environment Research Laboratories, Industrial Technology Research Institute (ITRI), Hsinchu 310, Taiwan; 4 Compound Semiconductor and Power Electronic System Division Electronic and Optoelectronic System Research Laboratories, Industrial Technology Research Institute (ITRI), Hsinchu 310, Taiwan; 5 Institute of Biomedical Engineering, National Yang Ming Chiao Tung University1001 University Rd., Hsinchu City 30010, Taiwan

**Keywords:** electroencephalography (EEG), electrophysiological biosensor, brain cognitive assessment, conductive hydrogel, polydopamine, silver nanowire (AgNW), self-healing

## Abstract

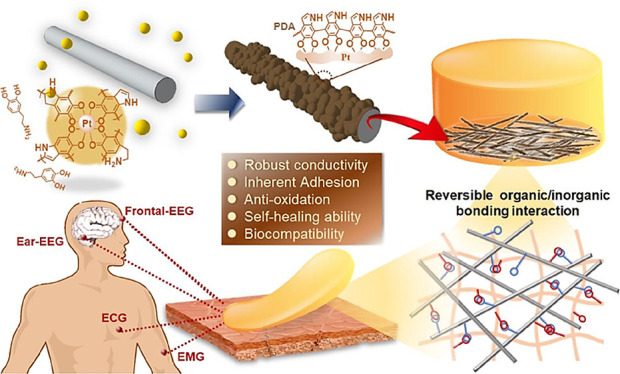

Continuous brain function monitoring by high-performance electroencephalogram (EEG) suggests a high impact for advancing precision personalized medication of neurodevelopmental or neurodegenerative disorders. Forehead and ear EEGs are nonhairy recording strategies that allow the recording of brain activity using only a few electrodes. However, they require well-designed electrodes that are easy and comfortable to carry while simultaneously performing durable high-quality EEG acquisition. Herein, we propose a new ultrabiocompliant EEG sensor that enables seamless contact to surfaces of both earhole and forehead, while permitting prolonged and high-quality EEG signal identification. Bioinspired polydopamine/platinum–silver nanowires, called PDA-Ag@Pt NWs, are synthesized with noticeable performances in electrical conductivity, antioxidation ability, cytocompatibility, and adhesion. PDA-Ag@Pt NWs can promote synchronic gelation and interlinks within polydopamine–polyacrylamide (PDA–PAM) hydrogels, in turn leading to the one-step formation of a nanowire/hydrogel matrix, called PDA–PAM/NW, as an electrode patch in the presence of adhesive and self-healing capabilities. Combined with a self-designed signal processor, a portable electrophysiological signal recording system was realized. The PDA–PAM/NW electrode patch outperformed commercial electrodes in terms of reliability and resolution for electrocardiography (ECG), electromyography (EMG), and electroencephalography (EEG) recording. In addition, through brain cognitive assessment by frontal- and ear-EEG recording, the ultrathin design and comfortable adhesion of PDA–PAM/NW electrodes make participants comfortable over time, subsequently providing the identification of the brain activity in high resolution. This work underscores the potential of the ultrabiocompliant and durable patch in the development of comfy, long-lasting, and high-performance wearable brain–machine interfaces for the revolution in neuroscience.

## 1 Introduction

Continuous physiological monitoring through high-performance wearable bioelectronic devices suggests a high impact for precision medicine in recent decades.^1^ Electroencephalograms (EEGs) are indicators of brain functions such as memory and cognitive states for early detection and treatment of neurodegenerative or neurodevelopmental disorders. However, EEG amplitude ranging from 0.5 to 100 μV is approximately 100 times smaller than other signals, including electrocardiography (ECG) and electromyography (EMG) (0.1–5 mV). The conventional strategy for the acquisition of high-quality EEG signals relies on a multichannel cap or a headband of a large size placing cross hairy scalp, which is inconvenient and nonpractical for patients in motion. Recently, nonhairy EEG including forehead or ear EEG allows to record the brain activity using only a few electrodes.^2,3^ A well-designed bioelectrode with high-performance EEG recording needs to provide comfortable adhesion with the earhole or forehead to continuously convert ionic currents into electron currents stably with a minimum impedance.^4,5^ However, currently in clinics, the mostly used electrodes are for scalp skin only, and most are wet electrodes composed of nondeformable metal caps or sheets that require coating with conductive gel to increase conductivity and facilitate tissue-device adhesion.^6−8^ These wet electrodes are time-consuming to use, and in addition, the gel dehydration occurring over time will gradually impair signal acquisition. Although dry electrodes can overcome the common challenges with wet electrodes, they cannot enable conformable and laminated contact with the forehead and earhole, causing a rise in electrode impedance, signal instability, and movement artifacts.

Recently, electroconductive hydrogels (ECHs) become attractive in the application of bioelectrode as they can provide a cell-habitable physical microenvironment to allow seamless tissue-skin lamination and free breathing of the skins, also exhibiting both remarkable ionic and electrical conductivity.^9,10^ ECHs are composed of two major components, i.e., hydrophilic polymer matrixes and electroconductive additives. In order to display tissue-mimicked softness, most ECH electrodes have been fabricated by embedding conductive polymer materials such as PEDOT,^11^ polyaniline (PANI),^12^ and polypyrrole,^13^ as well as semiconductor materials like carbon nanotubes (CNTs)^14^ and graphene.^15,16^ However, a major drawback of employing conductive polymers in ECHs is their lack of conductivity,^17−19^ while other issues that affect their long-term performance include biofouling, mechanical instability under dynamic conditions, and environmental instability. On the other hand, metallic materials offer high conductivity and stability but are less flexible and have a susceptibility to oxidative degradation, while organic conductors provide better flexibility and biocompatibility. Optimizing these trade-offs is key for advanced bioelectronics.^20^ Among metal nanomaterials, Ag nanowires (AgNWs) possess intrinsic durability in conductivity and one-dimensional geometry with a high aspect ratio allowing rich contact points with low resistance.^21,22^ Incorporation of Ag NWs in the hydrogel matrix can improve mechanical and electrical properties; thereby, the resulting ECH composites are applied for wearable biosensing in a high resolution in a wide detection range.^23,24^ Despite there being numerous research projects developing AgNW-based ECHs, Ag essentially has a high oxidation tendency when exposed to hydrophilic polymers, further imposing the risks of skin irritation and signal attenuation. Meanwhile, most AgNW-based ECHs have a deficiency of interlinks between metals and polymer networks that easily results in the loss of structural stability and conductivity, further limiting the application in long-lasting electrophysiological recording.

Polydopamine (PDA), the self-polymerized form of dopamine, has been receiving increasing attention in the development of organic bioelectronics. The predominant polymerized form is composed of the indole monomer, 5,6-dihydroxyindole (DHI), that presents naturally occurring melanin-like semiconductor properties and electron transfer ability because of rich π-electron mobility for carrier extractor and conductive junction.^25,26^ Meanwhile, PDA carries abundant catechol functional groups with remarkable free radical scavenging ability that contributes to robust antioxidation ability in a moisture-rich bio-microenvironment.^27^ The catechol group also plays a role as an active site, providing many types of bonding including covalent bonding, π–π stacking, metal chelation, or hydrogen bonding that can connect with a variety of substrate materials such as metals, semiconductors, ceramics, and synthetic polymers.^28^ More interestingly, in recent decades, it has been found that the switchable redox of catechol groups permits reversible adhesion of PDA, further endowing PDA-based composite materials with a controllable self-healable capability. Driven by reversible supramolecular bonding interactions between catechol groups and oxygen-rich functional groups of other polymers, many PDA-based hydrogels achieved approximately 75–95% healing efficiency in tensile strength and 95–97% in elongation at break after being cut and rejoined.^29−32^ PDA can provide a versatile solution for developing wearable bioelectronics.

**Scheme 1:**
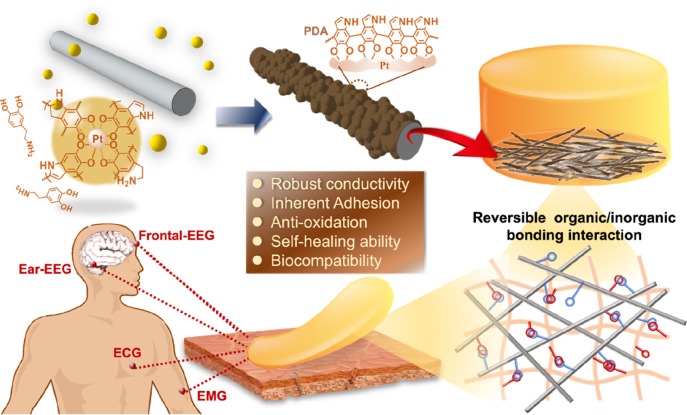
Bioinspired Polydopamine/Platinum–Silver Nanowires, Called PDA-Ag@Pt NWs, Are Synthesized with Robust Electrical Conductivity, Antioxidation Ability, Cytocompatibility, and Adhesion PDA-Ag@Pt NWs can promote synchronic gelation and interlinks within polydopamine–polyacrylamide (PDA–PAM) hydrogels, in turn leading to one-step formation of a nanowire/hydrogel matrix, called PDA–PAM/NW. The PDA–PAM/NW electrode patch outperformed commercial electrodes in terms of reliability and resolution for various electrophysiological signal recordings.

Herein, we introduce a new biopotential recording electrode-containing PDA-inspired technology including high conductivity, resistance to oxidation, biocompatibility, adhesion, and self-healing ability (Scheme 1). PDA/platinum-modified silver nanowires (PDA-Ag@Pt NWs) were synthesized following galvanic replacement reactions where PDA reduced and guided Pt to uniformly encapsulate Ag NWs, contributing to the formation of new NWs with robust conductivity, biocompatibility, antioxidant ability, and adhesion. On the other hand, PDA-Ag@Pt NWs promoted gelation and interlinking simultaneously within polydopamine–polyacrylamide (PDA–PAM) networks, leading to one-step formation of a hybrid electrode patch, called PDA–PAM/NW, exhibiting self-healing capabilities. The integration of heterogeneous systems was completed by combining a PDA–PAM/NW electrode and a homemade signal processor, further showing the superior performance in the electrophysiological signals including EMG, ECG, and EEG as compared with a commercialized electrode. Finally, through a cognitive function assessment, the PDA–PAM/NW patch exhibits the specific resolving power of forehead- and ear-EEG recording with only two channel sites, revealing potential perspectives in the application of neuroscience.

## 2 Materials and Methods

### 2.1 Materials

Ethylene glycol (EG, anhydrous, 99.8%), copper(II) chloride (CuCl_2_, 97%), polyvinylpyrrolidone (PVP, ACS Reagent, MW 55,000), silver nitrate (AgNO_3_, ACS Reagent, 99.0%), dopamine hydrochloride (DA), acrylamide (AM), potassium persulfate (KPS), *N*,*N*′-methylene bis(acrylamide) (BIS), acetone, tetramethylethylenediamine (TEMED), chloroplatinic acid solution (H_2_PtCl_6_), and sodium chloride (NaCl, ACS Reagent, 99.0%) were purchased from Sigma-Aldrich, Inc. (St. Louis, Missouri, USA).

### 2.2 Synthesis of Ag NWs and PDA-Ag@Pt NWs

The synthesis of Ag NWs was facilitated in oil bath using a hydrothermal method referred to a previous reference published by Korte et al.^54^ Briefly, 5 mL of EG was heated in an oil bath at 150 °C under magnetic stirring for 1 h. Next, 40 μL of a 4 mM CuCl_2_ solution was injected into the heated EG to form a homogeneous solution by stirring. Subsequently, 1.5 mL of 0.147 M PVP solution and 1.3 mL of 0.094 M AgNO_3_ solution were added, and then the reaction proceeded for another 1 h in the oil bath. The mixture was then transferred to a 50 mL Teflon-lined stainless steel autoclave and subjected to hydrothermal aging at 150 °C. After 1 h, the final product was naturally cooled down at room temperature. For purification, the as-synthesized sample was centrifuged at 2000 rpm for 20 min, and the supernatant was removed. The purified Ag NWs were finally dispersed in ethanol for further use. PDA-Ag@Pt NWs were synthesized via a combination of galvanic replacement reaction and self-polymerization. Briefly, 2 mL of the as-prepared AgNWs (30 mg/mL) were added to 1, 9.6, 9.2, and 8.8 mL of 1 mg/mL PVP solution, followed by addition with 0, 1.2, 2.4, and 3.6 mL of 300 mM dopamine (DA) solution to form four samples called Ag@Pt, PDA-Ag@Pt NW-1, PDA-Ag@Pt NW-2, and PDA-Ag@Pt NW-3, individually. After addition of 2 mL of 1 mM H_2_PtCl_6_ solution, the mixtures were stirred for 1 h. The resulting solutions were purified by centrifugation. The precipitate was redispersed in 20 mL of a 0.1 M NaCl solution and 20 mL of water, followed by centrifugation to collect the purified precipitate. The purifying procedure was repeated two to three times until the supernatant was fully clear. The final products were dispersed in water with a concentration of 5 mg/mL for further use.

### 2.3 Preparation of the PDA–PAM/NW Hydrogel Electrode Patch

The electrode patch was fabricated by combining PDA-Ag@Pt NWs with the PDA–PAM hydrogels. The PDA–PAM hydrogel was prepared by a modified method addressed by Han et al.^32^ Briefly, DA (2.5 mg/mL) was dissolved in 3 mL of a NaOH solution (at pH = 11) and allowed to self-polymerize for 20 min. Afterward, 0.5 g of AM, 40 mg of KPS, and 4 mg of BIS were then added and stirred in an ice bath for 10 min, followed by the addition of 2 μL of TEMED to enhance the gelation process. To make the PDA–PAM/NW patch, DA-Ag@Pt NW ethanol solution (2, 5, and 10 mg) was dropped on a polytetrafluoroethylene (PTFE) substrate with an enclosed area of 10 mm × 10 mm and dried at 50 °C for water evaporation. Then, 150 μL of the PDA–PAM precursor solution was dropped on the NWs and gelated for 30 min. Finally, the hybrid hydrogel membrane, PDA–PAM/NW, was peeled off.

### 2.4 Characteristics of NWs and Hydrogels

The morphology of various NWs was explored by SEM (JEOL-JSM6700) and Cryo-TEM (JEOL-JEM2010) at an accelerating voltage of 5 kV. In order to further analyze the elemental distribution within the NWs, energy-dispersive X-ray spectroscopy (EDX) with line scans was performed. The chemical composition characterization was conducted by X-ray photoemission spectroscopy (XPS) (Thermo Fisher Scientific Theta Probe) using monochromatic aluminum Kα X-radiation, which was operated in constant-pass energy mode. UV–visible spectroscopy was employed to evaluate the resistance to the oxidation of Ag NWs, Ag@Pt NWs, and PDA-Ag@Pt NWs. Each group of NWs was treated with H_2_O_2_ at concentrations ranging from 0.5 to 8 mM for 1 h. The UV–visible spectra were recorded over a wavelength range of 250 to 700 nm. FTIR spectroscopy (PerkinElmer Spectrum 100), operating in the wavenumber range of 450 to 4000 cm^–1^, was used to probe the intermolecular interactions of PDA–PAM and NWs. PDA–PAM and PDA–PAM/NWs in different thicknesses of NWs (*T* = 2, 5, and 12 μm) were applied for stress–strain tests using a microtensile tester (MTS, Tytron 250) with applied loads ranging from 0 to 50 N at a controlled rate of 0.1 mm/s. Adhesion properties were characterized using a texture analyzer (TA.XTplus Connect, Texture Technologies) in compression mode with a force of 3 g and a retraction speed of 10 mm/s. Hydrogel samples were prepared with dimensions of 10 mm in diameter and 0.5 mm in thickness and adhered to a glass substrate. The strain-resistance tests were conducted using a motorized linear stage connected to a digital multimeter (Keithley 2000) to monitor the resistance change. The swelling ratio of the hydrogels was estimated by measuring the change in the sample weight before and after immersion in deionized water. The procedure was repeated five times. The swelling ratio was determined according to the following equation:
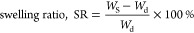
1where *W*_s_ and *W*_d_ represent the weights of the swollen and dried samples, respectively.

The resistivity was calculated using standard four-point probe methods (Jandel RM3000) (eq 1), and the conductivity of different NWs was determined according to the procedure outlined in eq 2.
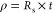
2
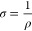
3where ρ is the resistivity, *R*_s_ is the sheet resistance, *t* is the thickness, and σ is the conductivity.^33^

The PDA–PAM gelation dynamics were indirectly measured using a rheometer (Discovery HR-1, TA Instruments, New Castle, Delaware, USA) in a cone geometry (diameter: 20 mm, angle: 1°). PDA–PAM precursor solutions were freshly prepared on the center of the plate with a nominal gap distance of 800 μm and measured by a linear time sweep under certain strain and frequency (γ = 1% and ω = 1 Hz) for 25 min. PDA–PAM with NW thickness (*T* = 12 μm) was prepared on a circle substrate (diameter = 2 cm) with a total 10 mm thickness. Storage (*G*′) and loss (*G*″) moduli were measured by a linear frequency sweep along with increasing strain amplitude (ω = 1 Hz, γ = 0.1–250%) or linear strain amplitude along with increasing angular frequency (γ = 1%, ω = 1–100 Hz). Impedance measurement was conducted for commercial Ag/AgCl and PDA–PAM/NW electrodes, respectively, by using the electrochemical 600E potentiostat/galvanostat instrument (CH Instruments, Austin, Texas, USA). Two electrodes, assigned as the reference and working electrodes, respectively, 2 cm apart are attached to the arm skin.

### 2.5 Self-Healing Properties of PDA–PAM and PDA–PAM/NW

The self-healing ability of the hydrogel was evaluated following the method outlined by Chen et al.^34^ An alternating strain scanning test was conducted at a constant frequency of 1 Hz to investigate the hydrogel recovery performance. The strain amplitude was cycled between 10 and 250% with a 600 s interval at each strain level. For the self-healing assessment, a gentle cut was applied on the hydrogel surface by using a razor blade at room temperature. The healing process was monitored over time, and the changes in the cut area and mechanical recovery were documented by using a camera to capture optical images throughout the experiment.

### 2.6 Cytotoxicity Assessment

Cytotoxicity was explored using CytoSelect Cell viability and cytotoxicity assay (Cell Biolabs, Inc., USA). PC- 12 cells (1 × 10^4^) were seeded per well of a 96-well plate. The plate was incubated for 24 h at 37 °C and 5% CO_2_ to allow cells to adhere and reach exponential growth. Different concentrations of the NW solution (50, 100, and 200 μg/mL) were prepared in a culture medium. The culture medium was removed from the wells, and 100 μL of each concentration was added to the designated wells. For controls, 100 μL of fresh culture medium (negative control) and DMSO as a cytotoxic agent (positive control) were added. The plate was incubated for 24 h. After the incubation period, the medium was carefully removed from each well without disturbing the cells. 100 μL of fresh culture medium and 10 μL of CCK-8 reagent (5 mg/mL in PBS) were added to each well. The plate was incubated for 3–4 h at 37 °C. The viability was determined by the Cell Counting Kit-8 (CCK-8) assay. The Live–Dead reagents include calcein-AM for staining the living cells (green) and ethidium homodimer-1 (EthD-1) to stain the dead cells (red). The cells were seeded in a well containing different concentrations of NWs (50, 100, and 200 μg/mL) with 5 × 10^4^ cells per well. After 24 h cell culture, the diluted Live–Dead reagents in PBS were directly added to the cell culture media at the ratio of reagent to culture media of 1:1, followed by a gentle mixing. After incubation in the dark for 30 min, five representative fields of view were selected per well under a fluorescent microscope. The numbers of live (green) and dead (red) cells were counted using the multiwavelength cell scoring module of MetaMorph software.

### 2.7 Electrophysiological Signal Recording

Commercial Ag/AgCl electrodes were used as a control for comparison. The long-term performance of the PDA–PAM/NW electrode is investigated by short-term tests under harsher conditions. The electrodes were placed in 2 M H_2_O_2_ for 1 month to measure the signal-to-noise (SNR) ratio. The electrophysiological data were acquired using a self-designed acquisition system as described in our previous study.^35^ This system was composed of an RISC MCU based on OpenRISC 1200 software developed using MATLAB 201, a programmable amplifier with changeable gains from 1 to 32×, and a multichannel preamplifier with a gain of 1000×. ECG was performed according to the Rauf et al. procedure.^36^ where a single-lead system was used to set up working and reference electrodes on each arm. According to Zhao et al. protocol,^37^ EMG measurements were performed with the reference electrode on the back of the wrist and the working electrode on the forearm. For EEG data acquisition, a total of eight recordings were conducted, split into memory and sudoku sets with four each, with four participants (two males and two females of the same ages). EEG signals were obtained from the forehead and ear to measure recognition and memory simultaneously by sticking electrodes on the forehead and into the earhole to get signals of the frontal (Fp1 and Fp2) and temporal (T3 and T4) lobes, respectively.

### 2.8 Statistics

All data were analyzed using MATLAB. Results are presented as the mean ± standard deviation (SD) from at least three independent experiments. The standard deviation represents a 95% confidence interval.

## 3 Results and Discussion

### 3.1 Preparation and Characterization of PDA-Pt@Ag NWs

**Figure 1:**
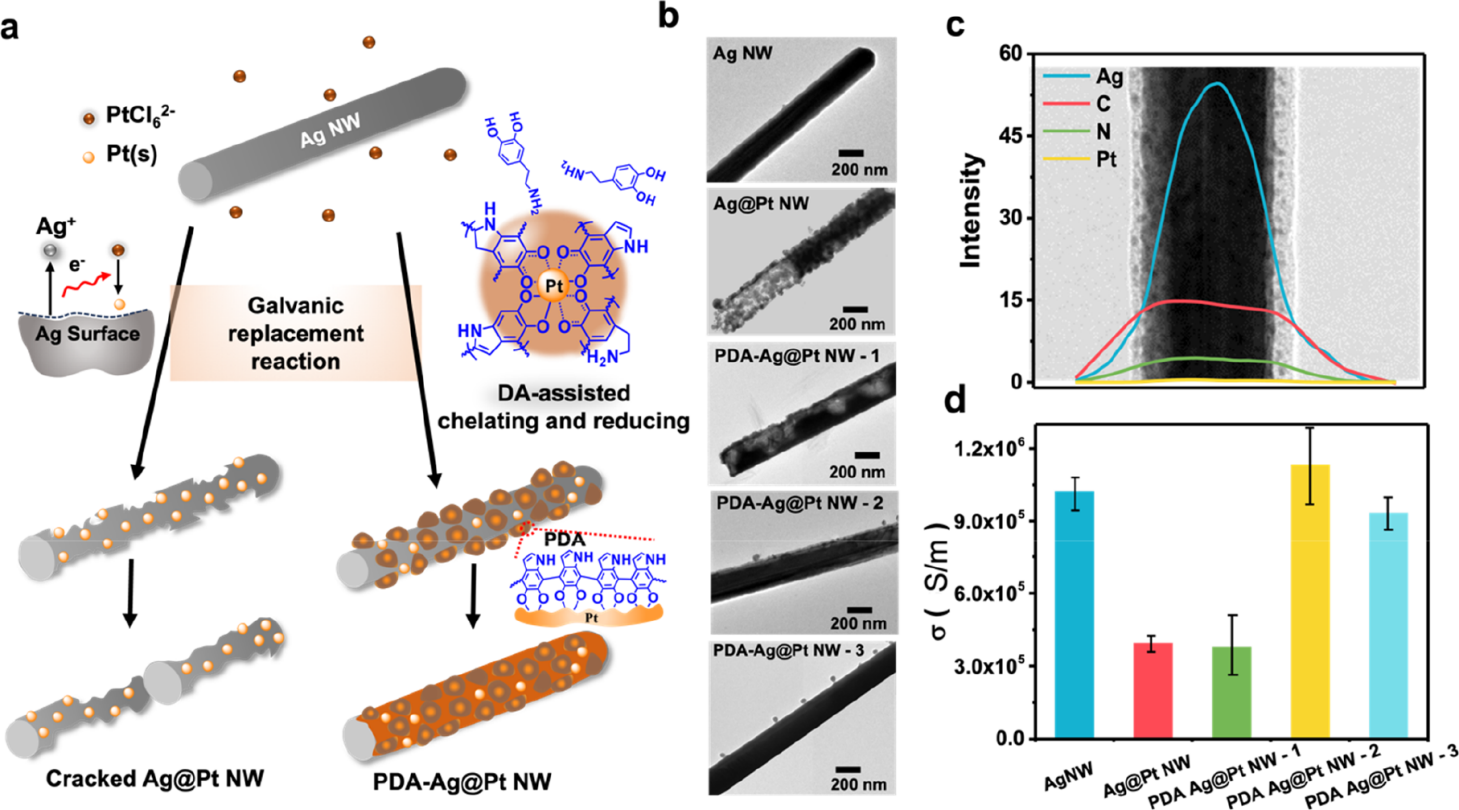
Formation and characterization of PDA-Pt@Ag NWs. (a) Schematic of the galvanic replacement reaction between Ag and Pt with DA enhancing structural integrity and conductivity. (b) TEM images show the smoother NW surfaces as the DA concentration is increased. (c) EDS mapping confirms Pt, carbon, and nitrogen presence. (d) Electrical conductivity of Ag NWs, Pt-coated NWs, and three PDA-Pt@Ag NWs.

PDA-Pt@Ag NWs were formed via galvanic replacement reaction occurring between Ag and Pt, and meanwhile, the incorporated DA is a reduction agent with surface and electroactivity permitting enhanced structural integrity and conductivity of final NWs. First, Ag NWs were synthesized by a hydrothermal process that facilitated the one-dimensional crystal growth of Ag to achieve a high yield of NWs with an average length of 78 ± 9 μm (Figure S1). As the galvanic replacement reaction proceeded, oxidation of the Ag atom accompanied by the reduction of Pt occurred randomly on the surfaces of NWs, which caused the formation of NW with rough surfaces and cracked interiors.^38^ Conversely, the incorporated DA offered a chelation force to PtCl_6_^2–^ and enhanced Pt reduction, while the self-oxidized form, PDA, enabled uniform deposition of Pt on Ag. It can be observed on the TEM images that as the DA concentration was increased, a well-defined smooth surface and high structural integrity were obtained for the resulting NWs (Figure 1b and Figure S2). PDA modification improved the cracked morphology, which is attributed to the antioxidation ability that resists Ag oxidation to Ag^+^. This alleviates the structural damage of the Ag nanowires. Meanwhile, PDA has rich amine and hydroxyl moieties grafting with charged Pt complexes, in turn permitting well-dispersed Pt nanoparticles to form a uniform Pt layer with high structural stability; thus, no cracks form in the interiors of Ag NWs.^39^ According to the result of EDS elemental mapping by TEM (Figure 1c), platinum, carbon, and nitrogen are found in the as-formed NW, proving that both Pt and PDA were successfully coated on the Ag NWs. The crystalline structures of the Ag NW and Pt@Ag NW were obtained by high-resolution TEM (HRTEM) (Figure S3). The lattice spacing of Ag NWs is calculated to be 0.235 nm, corresponding to the (111) plane of FCC crystalline Ag.^40^ The (110) plane of Ag is also shown with a lattice spacing of 0.290 nm. For Pt@Ag NW, Pt (111) was measured based on the lattice spacing value as 0.24 nm, and interestingly, Ag (200) was also found, which may be attributed to the existence of AgCl. There is no crystalline structure obtained from PDA-Ag@Pt NWs because of the PDA coating that covers the surfaces. To assess the chemical states of elements in the PDA-Pt@Ag NW, X-ray photoelectron spectroscopy (XPS) analysis was conducted. The wide-scan survey spectrum in Figure S3 presents the existence of C, O, N, Ag, and Pt, confirming the successful synthesis of PDA-Pt@Ag NWs. The observed peaks located at 368.3 and 374.3 eV in the high-resolution XPS spectrum are attributed to Ag 3d_5/2_ and Ag 3d_3/2_, respectively.^41,42^ This confirms that Ag remains in its elemental state without significant oxidation. Furthermore, the XPS peak of platinum (Pt) in the PDA-Pt@Ag NW system reveals characteristic peaks at 70.23 eV (Pt 4f_7/2_) and 73.55 eV (Pt 4f_5/2_), indicating the presence of metallic Pt. These binding energies align well with literature values for Pt^0^, suggesting the predominant existence of Pt in its elemental state.^43^ The absence of significant peak shifts toward higher binding energies implies minimal oxidation, confirming the stability and retention of the metallic nature of Pt within the PDA-Pt@Ag NW. PDA has a chemical structure resembling naturally occurring melanin exhibiting semiconductor properties; thereby, the as-formed PDA enhanced the charge transfer ability of NWs. The pristine Ag NWs displayed conductivity values of σ = 10.3 ± 0.6 × 10^5^ S m^–1^. Pt coating resulted in the reduction of conductivity to σ = 3.9 ± 0.2 × 10^5^ S m^–1^; however, for PDA-Ag@Pt NWs, the conductivity reached σ = 9.4 ± 0.6 × 10^5^ S m^–1^, which was comparable to that of Ag NW (Figure 1d).

### 3.2 Enhanced Oxidation Resistance and Biocompatibility of PDA-Pt@Ag NWs

**Figure 2:**
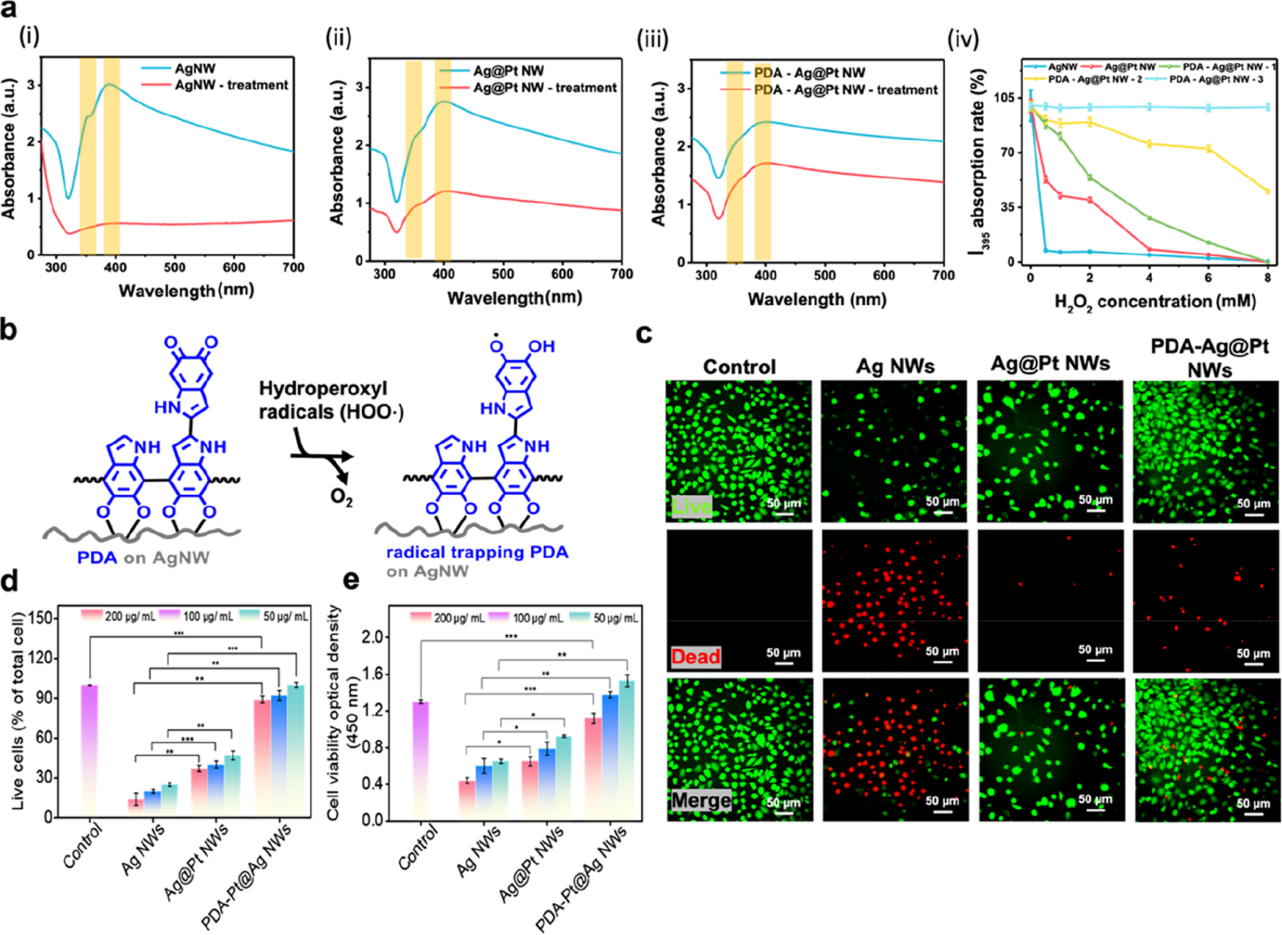
(a) UV–vis absorption spectra of Ag NWs, Ag@Pt NWs, and PDA-Pt@Ag NWs before and after H_2_O_2_ treatment. (b) Chemistry showing the radical trapping ability of PDA-Pt@Ag NWs. (c) Fluorescent images derived from the Live–Dead assay performed after 24 h. (d) Percentage of survived cells as calculated under a fluorescence microscope. (e) CCK-8 assay of cell viability after treatment with the samples at different concentrations for 24 h. All data were reported as mean ± standard deviation (SD) (*n* = 5).

PDA with excellent free radical trapping behavior contributed to the robust antioxidant capacity of the PDA-Pt@Ag NWs. H_2_O_2_, a main oxidant causing toxicity in living cells, was applied for investigating the resistance against oxidation. Figure 2a(i–iii) demonstrates the UV absorption spectrum of Ag NWs, Ag@Pt NWs, and PDA-Pt@Ag NWs, respectively. There are two main characteristic peaks of AgNW, namely, 350 and 395 nm, which are attributed to quadrupole resonance excitation mode and transverse plasma resonance mode, respectively.^44^ Upon treatment with H_2_O_2_, characteristic peaks of pristine Ag NW vanish due to the oxidation reaction. By contrast, Pt provides a protection shell for inhibiting oxidation of Ag NW, while PDA further minimizes the impact of H_2_O_2_ on Ag NWs (Figure 2a(i–iii)). When incubated in H_2_O_2_ with different levels, it is found from Figure 2a(iv) that the characteristic peaks of Ag@Pt NWs in 4 mM H_2_O_2_ completely disappear; however, the characteristic peaks of PDA-Pt@Ag NWs-3 remain consistent even when the H_2_O_2_ level attends to 8 mM. The result indicates that PDA enables a much stronger protective effect than Pt for Ag NWs. The mechanism of the antioxidant ability of PDA-Pt@Ag NWs is derived from the 1,2-benzoquinone moieties that are the hydrogen-bond donor allowing complexing to protonated H_2_O_2_, i.e., hydroperoxyl radical, HOO•, and released bioactive O_2_^45^ (Figure 2b).

Subsequently, to examine whether the antioxidant effects of PDA-Pt@Ag NWs could be exhibited directly in biology, cytotoxicity was further investigated by a LIVE/DEAD assay for PC-12 cells cultured with Ag NWs, Ag@Pt NWs, and PDA-Pt@Ag NWs. After 24 h cell culture, Figure 2c demonstrates the different live/death cell densities between each group. Compared with Ag NWs that significantly caused cell death, Ag@Pt NWs and PDA-Pt@Ag NWs allowed a higher proportion of survived cells, indicating that Pt and PDA improved the toxicity of Ag. Particularly, PDA-Pt@Ag NWs resulted in cell survival proportions comparable to those of the control group (Figure 2d). A similar tendency was observed from the results derived from the CCK-8 assay (Figure 2e), showing the robust cell viability of PDA-Pt@Ag NWs. These results reveal that PDA-Pt@Ag NWs play a role as biological antioxidants to protect cells against apoptosis.

### 3.3 Fabrication of the PDA–PAM/NWs ECH Electrode Patch

**Figure 3:**
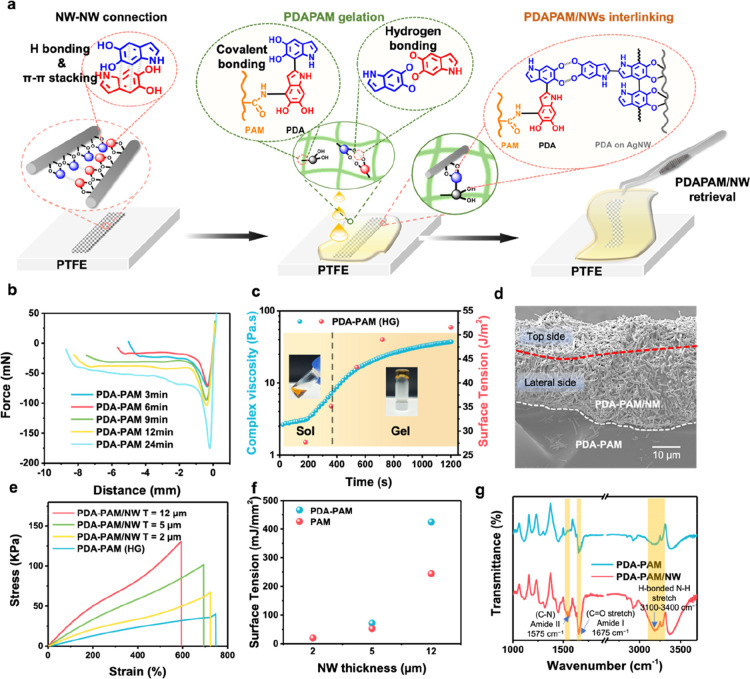
Fabrication and characterization of the PDA–PAM/NW ECH patch. (a) Schematic of PDA–PAM/NW fabrication on PTFE. (b) Sol–gel transition and adhesion behavior of PDA–PAM. (c) Adhesion force–distance curves. (d) Cross-sectional SEM image of PDA–PAM/NW. (e) Tensile properties with different NW thicknesses (2, 5, and 12 μm). (f) Calculated adhesion strength as a function of NW thickness. (g) FTIR spectra show enhanced hydrogen bonding and catechol interactions contributed by both PDA–PAM and NW.

Catechol motifs existing in both PDA-Pt@Ag NWs and PDA–PAM hydrogel can rapidly integrate these two heterogeneous materials to form an ECH patch, called PDA–PAM/NW. Fabrication of a PDA–PAM/NW patch follows three processes operated on a PTFE substrate (Figure 3a): (1) production of a thin layer composed of PDA-Ag@Pt NWs by solvent-evaporated deposition; (2) covering of the PDA–PAM precursor on the deposited NWs for PDA-promoted sol–gel transition and adhesion; (3) produced interconnection between NWs and hydrogels, which enhances device integration. Catechols in PDA–PAM hydrogel precursors can oxidatively polymerize into networks through sol–gel transitions supported by increased adhesion. The adhesion of PDA–PAM at different time intervals of gelation was demonstrated in the recording of force–distance curves in Figure 3b. The kinetics of hydrogel formation and enhanced adhesion show that PDA–PAM produced hydrogels that can be handled (from 2.5 to 40 Pa·S) and exhibit adhesion strengths (from 48.5 to 51.5 J/m^2^) in approximately 20 min (Figure 3c). The cross-section view of the SEM image of PDA–PAM/NW displays a two-layer architecture including a NW-rich hydrogel layer and a pure hydrogel layer, respectively (Figure 3d). Tensile tests were performed on PDA–PAM/NW with different thicknesses of NW layers (2, 5, and 12 μm, respectively). Referring to the stress–strain curves in Figure 3e, as compared with the pristine PDA–PAM networks showing a maximum ductility of EL%_max_ = 736 ± 12%, the PDA–PAM/NW with a NW thickness of 12 μm can still preserve a ductility of 600% but show about threefold increase in Young’s modulus and tensile strength. Interestingly, PDA–PAM/NW exhibited self-adhesion dependent on the increased thickness of the NW layers (Figure 3f). The result is attributed to the formation of hydrogen bonding interactions proved by FTIR analysis. As shown in Figure 3g, the combination of PDA–PAM and NWs produces notable growth in amide I (−C=O, 1675 cm^–1^) and amide II (−C–N, 1575 cm^–1^), which is attributed to the catechol groups of PDA derived from the NWs. In addition, the enhancement of peaks at 3200–3400 cm^–1^ was attributed to the hydrogen-bonded N–H, suggesting that the rich catechol groups from both PDA–PAM and NWs resulted in enhanced hydrogen bonding and catechol coupling interactions. These forces are largely responsible for the mechanical robustness and adhesion of PDA–PAM/NW hydrogels.

### 3.4 Electrically Conductive Stability of PDA–PAM/NWs

**Figure 4:**
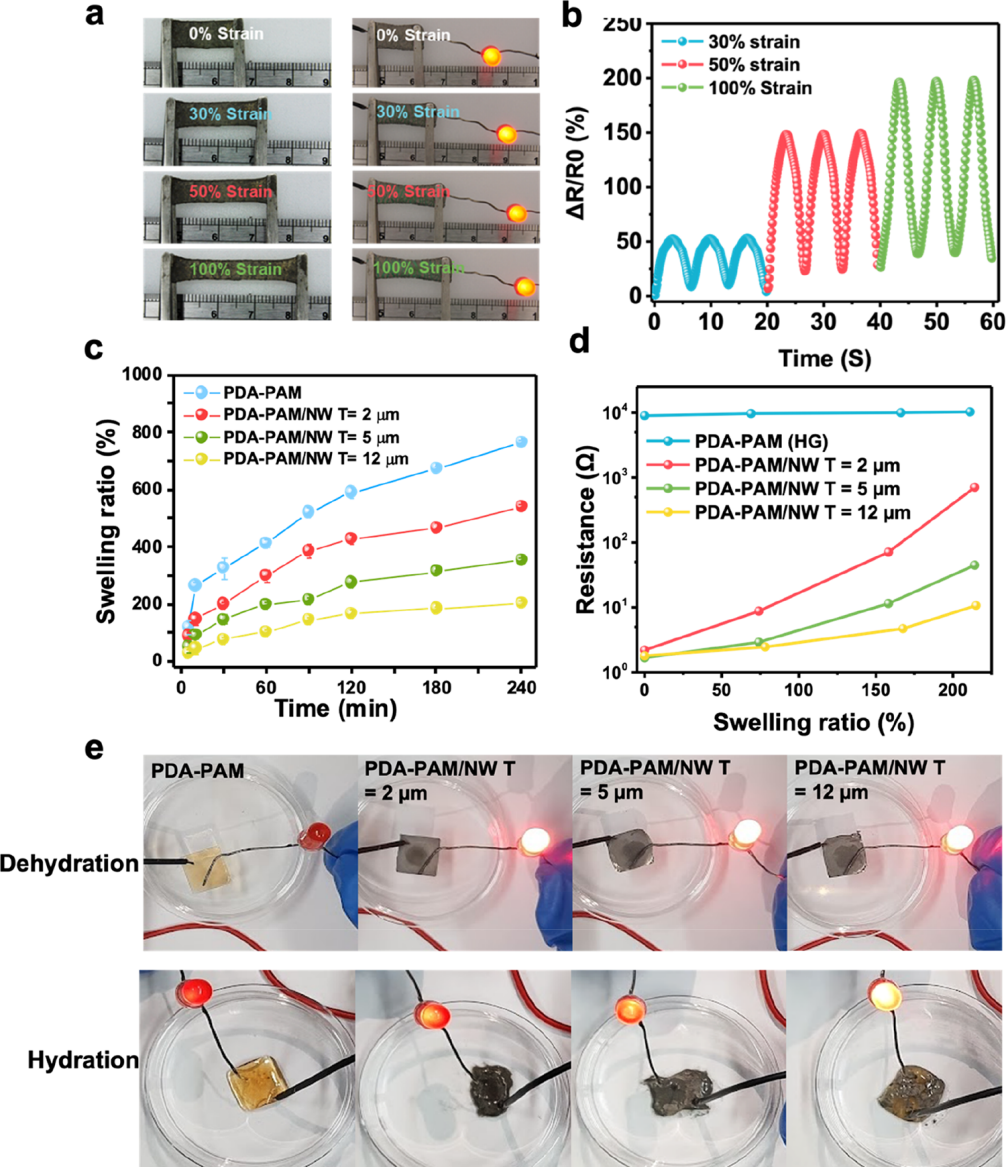
(a) Mechanical deformation test of PDA–PAM/NW under increasing strain levels, as shown by illuminated LED. (b) Cyclic resistance response of PDA–PAM/NW material under various stretchabilities of 30, 50, and 100%. (c) Moisture-dependent swelling of PDA–PAM/NW hydrogels with varying thicknesses of NW layers. (d) Electrical conductivity of PDA–PAM/NW hydrogels at different swelling states as a function of NW layer thickness. (e) Visual demonstration of electrical conductivity using LED bulbs for PDA–PAM/NW hydrogels with different NW layer thicknesses in a dry state and at a 200% swelling ratio.

The mechanical properties of PDA–PAM/NWs were demonstrated under various strain conditions, as shown in Figure 4a. The PDA–PAM/NW maintains its structural integrity even when stretched to double its original length (100% strain). The progressive strain test reflects the PDA–PAM/NW’s ability to endure the tensile stress while retaining functionality, as evidenced by the illuminated LED that remains light throughout the stretching process, confirming the material’s consistent electrical conductivity despite mechanical deformation. The electrical resistance change ratio, often termed cyclic resistance response during different strain conditions, is depicted in Figure 4b. The electrical resistance of the PDA–PAM/NW composite exhibited a gradual increase with increased strain, indicating a slight structural fatigue. However, at 30% stretchability, which is at least required for wearable applications, the resistance remained relatively stable, suggesting that PDA–PAM/NW is a promising candidate for stretchable electronic applications.

ECHs with consistent conductivity in a moisture-rich environment can provide stability of signal transduction in wearable utilization. Figure 4c displays the swelling behaviors of PDA–PAM/NW with different thicknesses of the NW layers. Catechol groups of both PDA–PAM and NMs act as cross-linking points; thereby, the thicker NW layer led to higher cross-links, in turn resulting in the decrease of both the swelling rate and equilibrium swollen ratio (ESR). The ESR of pure PDA–PAM hydrogels is 794 ± 23%, while PDA–PAM/NW with the thickness of NW layer = 12 μm presents an ESR of only 210 ± 16%. The electrical conductivity of each hydrogel at different swelling states is shown in Figure 4d. The thickness of the NM layer directly affected the conductive stability of hydrogels upon swelling. With the same swelling ratios, a straightforward result was found that PDA–PAM/NW with a thicker NW layer presented higher conductivity. Particularly, a hydrogel with the thickness of NW layer = 12 μm can maintain the resistance of 6.2 ± 1.2 ohm when its swelling ratio reaches 200%, showing the electrical reliability of the hydrogel under a moisture-rich physiological environment. Subsequently, the electrical conductivity of the hydrogels was visualized by connecting them with a light-emitting diode (LED) bulb (Figure 4e). At the dry state, all PDA–PAM/NWs show similar lightness. Upon the swelling ratio being increased up to 200% defined as the hydration state, PDA–PAM/NW with the highest thickness, i.e., 12 μm, displayed the highest brightness as compared with other samples.

### 3.5 Self-Healing Ability of PDA–PAM/NWs

**Figure 5:**
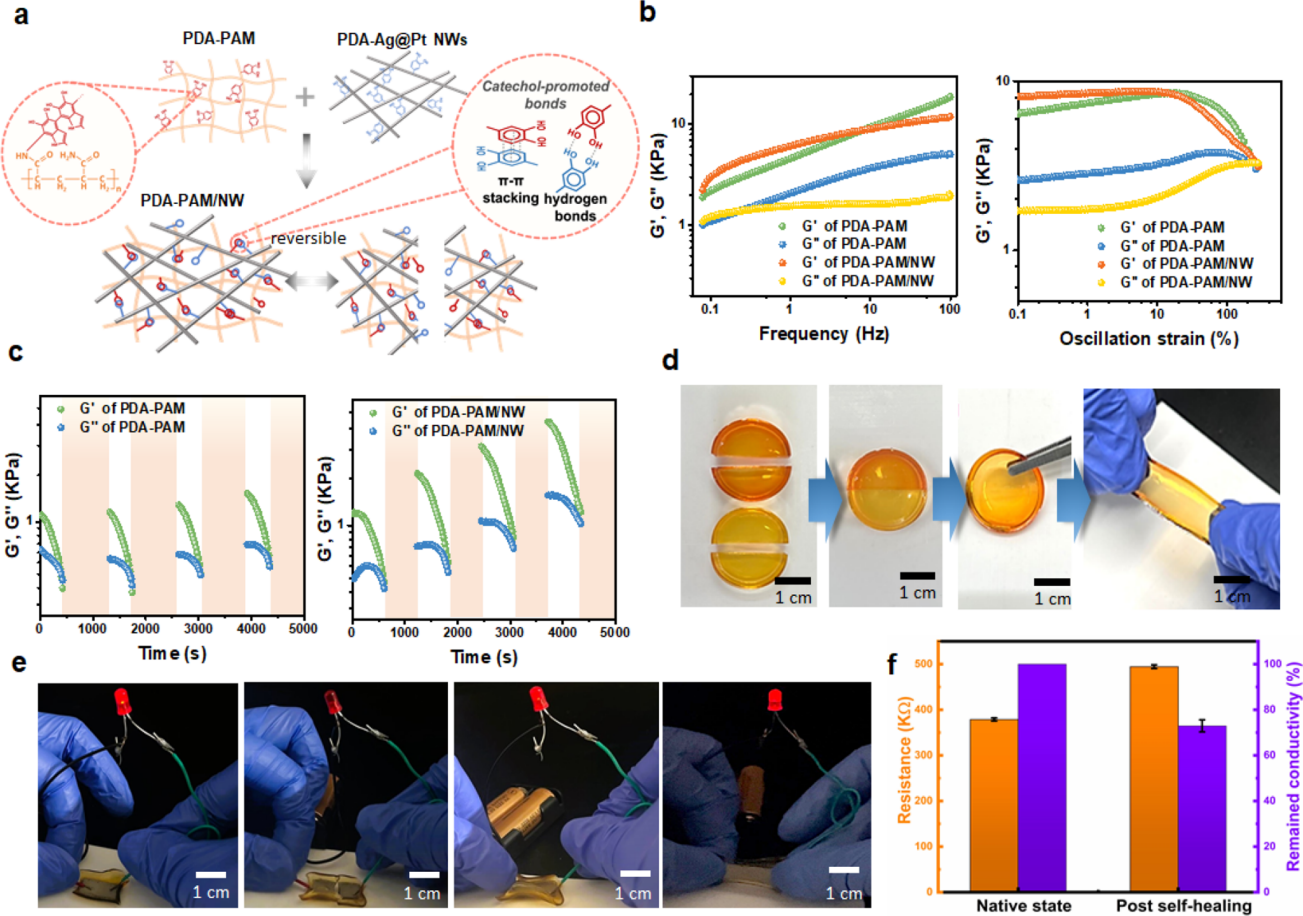
Self-healing properties of PDA–PAM/NW hydrogels. (a) Schematic illustration of the reversible bonds including hydrogen bonds, π–π stacking, and metal–organic chelation in PDA–PAM/NW hydrogels. (b) Frequency sweep rheological behavior of PDA–PAM and PDA–PAM/NW hydrogels under 0.1% strain, showing storage modulus (*G*′) and loss modulus (*G*″). (c) Alternating stepwise strain scanning demonstrating the stability and reconstruction ability of PDA–PAM and PDA–PAM/NW hydrogel networks. (d) Macroscopic self-healing demonstration: two differently colored PDA–PAM/NW hydrogels (red and green) merging within 30 min, with dye diffusion across the rejoined interface and maintained integrity under tensile force. (e) Self-healing and stretchable properties of PDA–PAM/NW hydrogel: original, cut, healed, and stretched states; the illuminated bulb confirms consistent electrical conductivity. (f) Comparison of electrical properties of PDA–PAM/NW hydrogel (native state) and post self-healing.

The supramolecular interactions between PDA–PAM and NWs including hydrogen bonds, π–π stacking, and metal–organic chelation endow the PDA–PAM/NW hydrogel with robust self-healing ability (Figure 5a). The rheological behavior investigated by frequency sweep under a strain of 0.1% is demonstrated in Figure 5b, where the storage moduli *G*′ of both PDA–PAM and PDA–PAM/NW are higher than the loss modulus *G*″ within the linear viscoelastic range, indicating that both hydrogels behaved similarly to elastic solids.^5^ Next, by rheological sweep over a range of strain at 1 Hz, it is observed from the intersection point of *G*′ and *G*″ at a strain of 125% that the hydrogels collapsed.^5^ In order to confirm the self-healing ability, alternating stepwise strain scanning from 50% through 150% was performed to evaluate the stability and reconstruction ability of the hydrogel networks (Figure 5c). In this case, the *G*′ values of both PDA–PAM and PDA–PAM/NW decreased sharply from 8.5 to 3.1 kPa, resulting in a quasi-liquid system with loss factor, tan δ = *G*″/*G*′ ≈ 1. After the subsequent self-healing time, the *G*′ values returned to the initial values, and the system returned to a gel state (tan δ < 1). Interestingly, both the *G*′ and *G*″ of PDA–PAM/NW showed a gradual increase in response to each sweep, which might be due to increased adhesion caused by the shear-induced formation of hydrogen bonds between the catechol groups.^6^ The macroscopic self-healing property is further observed from the merging of two pieces of PDA–PAM/NW hydrogels in different colors (red and green, respectively). Within 30 min, the two pieces merge with dye diffusion through the rejoined interfaces. The healed hydrogel could maintain integrity even after being applied with an external tensile force (Figure 5d). The reversible bonds from the PDA chains linked to the PAM network could dissipate energy efficiently through breakage of the noncovalent bonds to prevent crack propagation during stretching, therefore enhancing the toughness and increasing the extension ratio as well as endowing the hydrogel with excellent self-healing ability. Such a property is also visible in the conductivity. Figure 5e demonstrates electrical conductivity restoration through a connected LED bulb during the self-healing process of the PDA–PAM/NW hydrogel. The red LED remains lit before cutting, temporarily turns off when the material is separated, and successfully illuminates again after the hydrogel self-heals, providing visual proof of the restored electrical pathways. Figure 5f shows an approximately 73% conductivity retention after healing with measurements taken from three hydrogel samples, indicating consistent self-healing performance across the samples.

There are two self-healing interfaces, namely, (PDA–PAM)/(PDA–PAM) and (PDA–PAM)/NW interfaces, in the PDA–PAM/NW hydrogel electrode. The self-healing ability and mechanism of PDA–PAM hydrogels have been highly reported.^31,46^ Regarding the self-healing mechanism between PDA–PAM and NWs, the PDA-functionalized NWs (PDA-Ag@Pt NWs) possess rich functional groups (i.e., −NH_2_ and −OH) that can form reversible hydrogen bonding interactions with PDA–PAM. In addition, Li et al. performed molecular dynamic simulation, demonstrating the self-healing interface between PDA and OH-rich hydrogels based on the reversible hydrogel bonds.^47^

### 3.6 Electrophysiological (ECG, EMG, EEG) Monitoring

Electrophysiological detection for various signals including ECG, EMG, and EEG was applied for both PDA–PAM/NW and commercialized Ag-AgCl electrode studs for comparison. For impedance measurement, two electrodes, assigned as the reference and working electrodes, respectively, 2 cm apart, were attached to the skin of the arms. As shown in the Bode plot in Figure S4, the PDA–PAM/NW electrode placed on the skin exhibited a lower impedance than commercial Ag/AgCl electrodes within all frequency ranges (1 Hz to 10^5^ kHz). Particularly, at 1 kHz, the PDA–PAM/NW electrode showed an impedance value of 6.7 kΩ, which is similar to the values of wet electrodes reported previously.^48,49^

**Figure 6:**
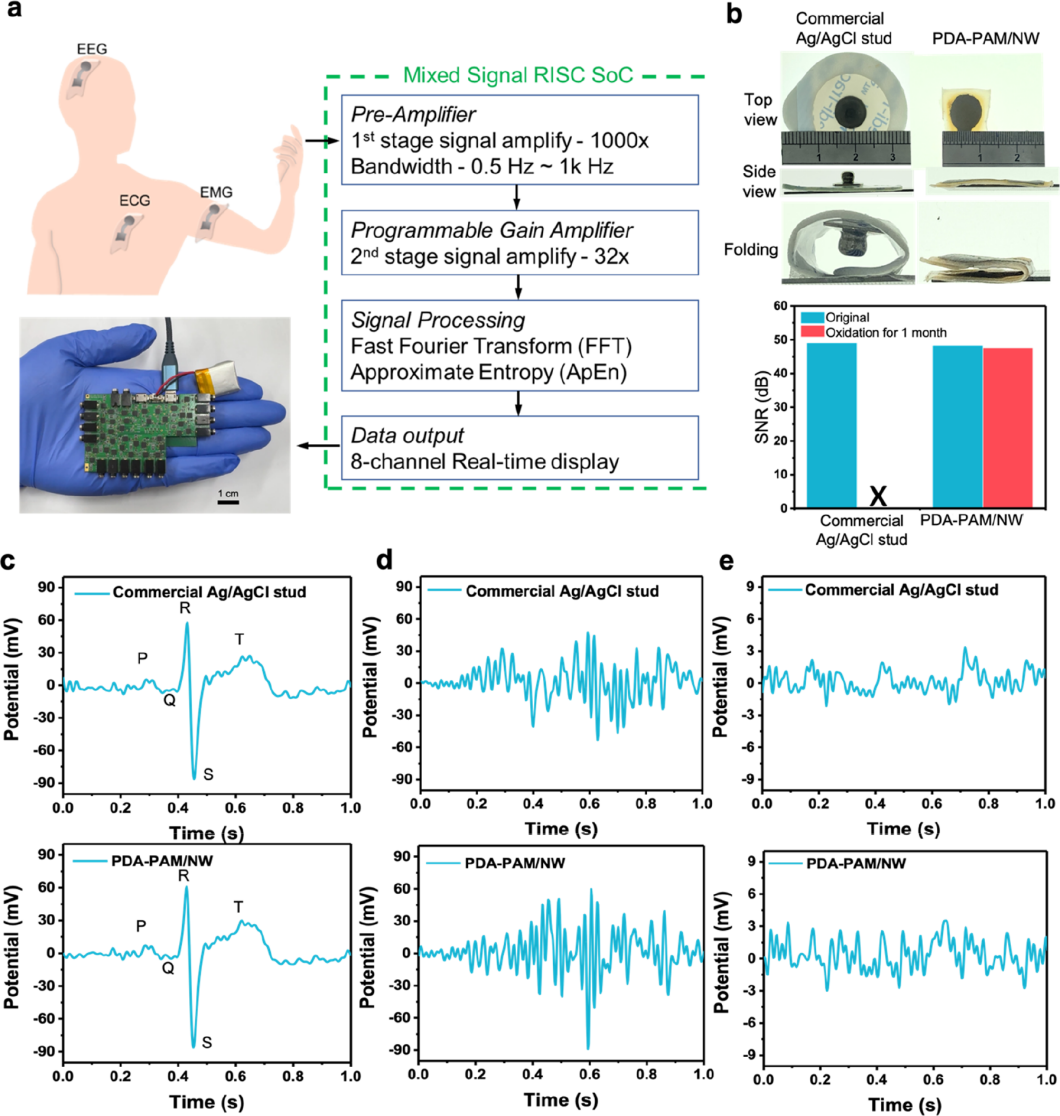
(a) Schematic and an optical image of the portable biosignal recording system with Mixed Signal RISC SoC, two-stage amplification, and onboard signal processing. (b) Comparison of conformable contact and SNR between PDA–PAM/NW and a commercial Ag-AgCl electrode, fresh and after 1 month of H_2_O_2_ exposure. (c) ECG waveforms, clearly identifying the P wave, QRS complex, and T wave, validating the PDA–PAM/NW electrode accuracy. (d) sEMG signals, demonstrating enhanced motor unit potential (MUP) detection with PDA–PAM/NW compared to commercial electrodes. (e) EEG recordings show consistent voltage oscillations, with PDA–PAM/NW capturing slightly higher-amplitude variations, indicating improved signal sensitivity.

To ensure the convenience for the detection of EEG, which has a relatively smaller signal amplitude (in the range of 0.0002–0.1 mV) than ECG and EMG (0.1–5 mV), we designed a portable biosignal recording system (Figure 6a). Particularly, the Mixed Signal RISC SoC architecture enhances portable biosignal processing, supported by a two-stage amplification process comprising a preamplifier (1000 × gain, 0.5 Hz–1 kHz bandwidth) and a programmable gain amplifier (32× gain) to flexibly accommodate diverse physiological signal amplitudes. Advanced onboard signal processing, including FFT and approximate entropy (ApEn), enables real-time complex analysis, crucial for rapid interpretation in clinical settings. As compared with commercial devices, the foldable, thin-film structure of the PDA–PAM/NW electrode reveals a conformable contact between the sensor and skin (Figure 6b)*.* The calculated SNR show that both devices have similar resolution. After being placed in 2 M H_2_O_2_ for 1 month, the commercial device fails while the PDA–PAM/NW can maintain the SNR (Figure 6b). The result is attributed to the robust antioxidant ability of PDA–PAM/NW, which thus permits a better environmental tolerance for long-term application. Figure 6c shows the recorded ECG waveforms over a 1 s interval. The ECG data including the P wave, QRS complex, and T wave obtained from the PDA–PAM/NW aligns closely with that from a commercial ECG device, validating its precision and reliability. Both electrodes were employed for surface electromyography (sEMG) on the forearm (Figure 6d and Figure S5), which detects motor unit potential (MUP) within a 20–2000 Hz bandwidth, and PDA–PAM/NW is more sensitive to vibrations and muscle contractions. The higher amplitude in MUP derived from the PDA–PAM/NW can be attributed to its better compliance with skin tissue. Figure 6e shows the recorded EEG data over a 1 s interval obtained from both electrodes. Both signals display rapid oscillations typical of EEG recordings, indicating ongoing brain activity. The commercial EEG shows voltage fluctuations between −2 and 3 mV, with occasional peaks slightly beyond this range. In contrast, the PDA–PAM/NW electrode captures similar EEG patterns but with slightly larger amplitude variations, ranging from −3 to 3.5 mV. The ability of PDA–PAM/NW to detect slightly more pronounced potential changes suggests it may offer enhanced sensitivity. The results highlight the potential of the PDA–PAM/NW electrodes as a promising tool for a variety of human-machine interfaces.

### 3.7 Cognitive Function Assessment

**Figure 7:**
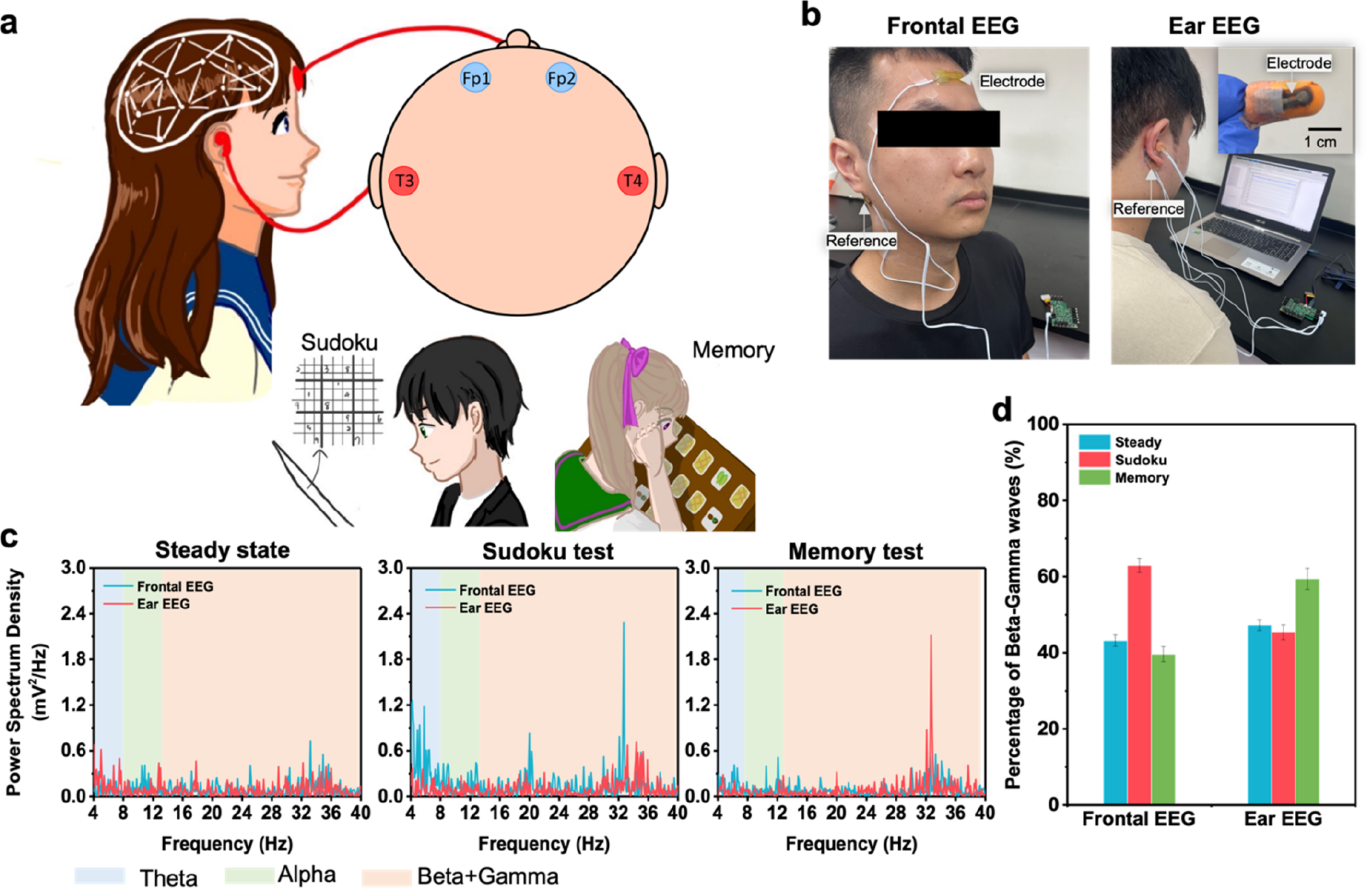
(a) Illustration of the placement positions of frontal and ear EEG electrodes to assess sudoku and memory tasks. Fp1 and Fp2 are for left and right frontal pole EEG electrodes, respectively, and T3 and T4 are for the left and right ear-EEG electrodes. (b) Images showing the experimental setup. (c) Power spectral density plots were calculated from the EEG data recorded at the state of resting, sudoku game assessment, and memory game assessment. (d) Percentage of beta–gamma waves derived from frontal and ear EEG recording in various cognitive tasks.

Assessment of the cognitive function responsible for brain activity requires an ideal EEG sensor for the identification of suitable EEG frequency bands and spatial locations. However, due to the inherently low signal strength (typically in the μV range), sustained EEG monitoring exhibits substantial challenges. Here, frontal- and ear-EEG recording using PDA–PAM/NW electrodes together with a portable recording system can make participants comfortable when performing cognitive tasks, including Sudoku puzzles and memory card games. Due to the ultraconformable and adhesion properties, the PDA–PAM/NW electrodes permit stable EEG recording over time. For data acquisition, two PDA–PAM/NW electrodes were positioned on the frontal (Fp1) and temporal (T4) lobes for frontal and ear EEGs, respectively, while a reference electrode was placed behind the ear (Figure 7a,b). The power spectral density (PSD) plots in Figure 7c indicate notable differences in EEG power distribution across the alpha (8–13 Hz), beta (13–30 Hz), and gamma (above 30 Hz) bands depending on the tasks. Specifically, as compared with the resting state, brain responses to the sudoku and memory tasks were presented by a remarkable increase in beta and gamma band power, reflecting the heightened cognitive demands and increased mental workload associated with these activities. Figure 7d illustrates the combined percentage of beta and gamma waves recorded from two different positions. For the steady state, which is typically associated with relaxation and minimal cognitive engagement, both the frontal and ear EEGs show similar popularity of beta and gamma waves. However, during the sudoku task, a remarkable increase in beta- and gamma-wave activity was recorded from the forehead. On the other hand, in the memory task, the earhole measurement demonstrated more increments in beta and gamma waves as compared to those obtained from the frontal measurement. The comparative analysis between the forehead and earhole placements reveals that Fp1 and Fp2 areas of the prefrontal lobe participate when subjects concentrate on problem-solving and cognitive activity, while T3 and T4 exhibit higher activity as the memory load is increased. The wave power shows varied trends across different tasks, thereby providing insights into the modulation of neural activity under different cognitive conditions. The comparison presented in Table 1 highlights the significant advancements of the PDA–PAM NW hydrogel electrode over previously reported bioelectrodes. Our electrode demonstrates a superior balance of high conductivity, long-term stability, self-healing capacity, and application. This suggests potential applications in selecting electrode placement for specific cognitive monitoring tasks, which can provide a comprehensive understanding of the brain’s dynamic response to varying mental activities.

**Table 1:** Comparative Analysis of Conductive Hydrogels for High-Performance Wearable Bioelectrodes

materials	conductivity (S/m)	contact impedance	long-term stability	self-healing ability	application	references
PEDOT:PSS hydrogel	106.53	25.47 Ω at 1 kHz	excellent	no	EMG	(11)
Ag nanowire (AgNW) hydrogel	1.3	N/A	high	no	ECG, wearable device, and motion detection	(21)
DA-PPy/PVA	0.06	N/A	high	no	ECG, EMG, wearable sensor, motion detection	(13)
PEDOT:PSS	N/A	72 KΩ at 50 Hz	excellent	no	ECG	(50)
HPMC/PVA hydrogel	7.26	<8 Ω	high	no	ear-EEG	(51)
PEDOT:PSS	0.745	<0.4 kΩ, 15 KΩ for dry electrode	high	no	EEG monitoring, BCI, neurophysiological monitoring	(52)
PVA/PVP/PDA-NP	N/A	3– 4 kΩ	high	no	EEG, BCI, neurophysiological monitoring	(53)
PDA-Ag@Pt NW PDA–PAM hydrogel	4.2 ± 1.1	6.7 kΩ	high	yes	ECG, EMG, and EEG	this work

## 4 Conclusions

In this work, a PDA–PAM/NW hydrogel composite was developed to enhance electrophysiological signal monitoring through improved electrical, mechanical, and biocompatible properties. The DA-inspired technology including semiconductive property, antioxidation, biocompatibility, and adhesion promoted the revolution of silver nanowires, leading to the formation of new organic–inorganic nanowires (PDA-Ag@Pt NWs) facilitating inter- and intralinking with polymer chains. The combination of the PDA-Ag@Pt NW with a PDA–PAM matrix successfully integrated the advantages of conductive nanomaterials and self-healing hydrogels, leading to a biocompatible, highly conductive, and conformal electrode patch. Our results demonstrated that the PDA–PAM/NW electrodes provided superior oxidative stability, enhanced adhesion, and mechanical robustness, which allowed for long-term use in wearable bioelectronics, outperforming commercial electrodes. Moreover, the PDA–PAM/NW electrodes showed excellent self-healing properties due to the dynamic supramolecular interactions between catechol groups, ensuring structural and functional recovery after damage. Electrophysiological tests confirmed that the PDA–PAM/NW patch effectively captured high-quality ECG, EMG, and EEG signals, exhibiting better sensitivity and signal-to-noise ratios in both short-term and long-term monitoring compared with conventional electrodes. Particularly, the EEG recordings during cognitive assessments highlighted the patch’s ability to resolve distinct brainwave patterns with improved resolution, offering significant potential in neurological and brain function studies. The integration of PDA-Ag@Pt NWs into a PDA–PAM matrix represents a promising strategy for developing next-generation human–machine interfaces with applications in continuous healthcare and diagnostics.

## Supporting Information Available

Comparisons of AgNW synthesis methods, morphology, and structural evolution of AgNW, Ag@Pt NW, and PDA-Ag@Pt NW are analyzed using SEM and TEM,respectively. HRTEM examines the crystalline structures of AgNW and Pt@Ag NW, while XPS confirms the chemical state of PDA-Ag@Pt NWs. Contact impedance measurement of wearable electrodes and enhanced sEMG evaluations for sEMG are included (PDF)
Gentle cut applied on the hydrogel surface by using a razor blade at room temperature (MP4)
