# Supplementary material for: Ultrabioconformal, Self-Healable, and Antioxidized Polydopamine-Inspired Nanowire Hydrogels Enable Resolving Power in Forehead and Ear Electroencephalograms for Brain Function Assessment
Source: ACS Appl Mater Interfaces. 2025 Apr 17;17(17):24887–900. doi: 10.1021/acsami.4c23013 (PMC12051168; doi:10.1021/acsami.4c23013)
Supplement: Supplementary file 1 — am4c23013_si_001.pdf [file am4c23013_si_001.pdf]

Supporting Information

Ultra-bioconformal, Self-healable, and Anti-oxidized Polydopamine-Inspired Nanowire Hydrogels Enable Resolving Power in Forehead- and Ear-Electroencephalogram for Brain Function Assessment

Kanishk Singh[1†], Chun-Chang Lin[1†], Wei-Han Huang [1], Wan-Lou Lei [1], Yu-Han Wang [2], Po-Hsueh Chang[3], Ru-Zheng Lin[4], Her-Ming Chiueh [1], Wei-Chen Huang[1,5*]

[†] K. Singh and C.-C. Lin equally contributed as the first authors of this paper.

[1] Department of Electrical and Computer Engineering, National Yang Ming Chiao Tung University 1001 University Rd., Hsinchu City 30010, Taiwan (R.O.C.)

[2] The Affiliated Senior High School of National Taiwan Normal University, No. 143, Sec. 3, Xinyi Rd., Taipei City 106348, Taiwan (R.O.C.)

[3] Low Carbon and Energy Storage Division, Green Energy & Environment Research Laboratories, Industrial Technology Research Institute (ITRI), Hsinchu, 310, Taiwan (R.O.C.)

[4] Compound Semiconductor and Power Electronic System Division Electronic and Optoelectronic System Research Laboratories, Industrial Technology Research Institute (ITRI), Hsinchu, 310, Taiwan (R.O.C.)

[5] Institute of Biomedical Engineering, National Yang Ming Chiao Tung University, 1001 University Rd., Hsinchu City 30010, Taiwan (R.O.C.)

Corresponding author:
Prof. W.-C. Huang,
Department of Electrical and Computer Engineering,
Institute of Biomedical Engineering,
National Yang Ming Chiao Tung University
1001 University Rd., Hsinchu 30010, Taiwan
*E-mail: weichenh@nycu.edu.tw

1. Comparison of AgNw Synthesis Methods: Oil Bath vs. Hydrothermal Process

Comparison of AgNw synthesis using two methods: the oil bath method at 2 h and 6 h, which showed suboptimal growth with spherical byproducts, and the hydrothermal process, which produced longer and more uniform AgNw structures, demonstrating improved synthesis efficiency.

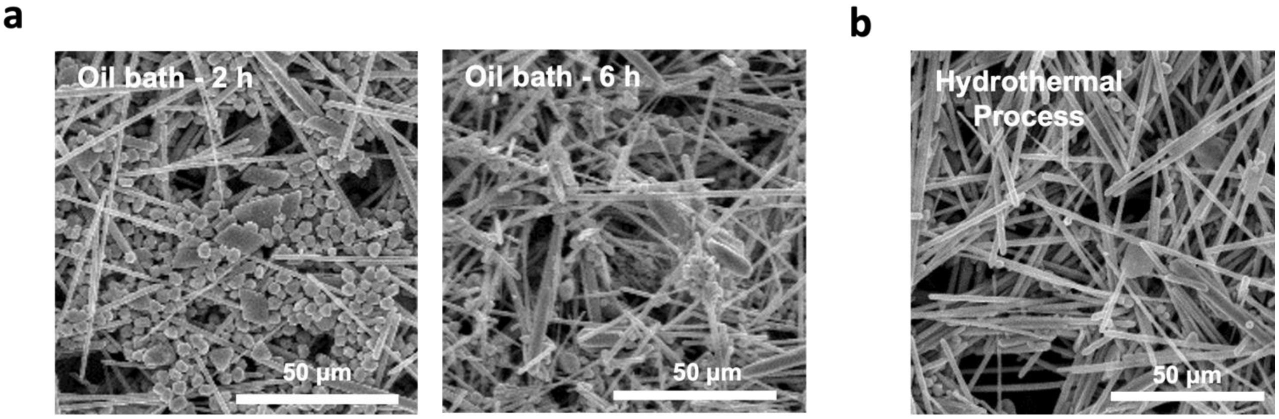

Figure S1 Comparison of AgNw synthesis methods. (a) Oil bath method at 2 h and 6 h, showing suboptimal growth with the presence of spherical particles, especially at 2 h. (b) Hydrothermal process, demonstrating improved AgNw with longer, more uniform structures

2. Structural Characterization of AgNW and Modified Ag@Pt NWs

Morphology and structural evolution of AgNW, Ag@Pt NW, and PDA-Ag@Pt NW using SEM and TEM imaging. SEM images illustrate the modification stages, while TEM reveals the core-shell structure, with an enlarged view emphasizing the PDA-Ag@Pt NW surface features.

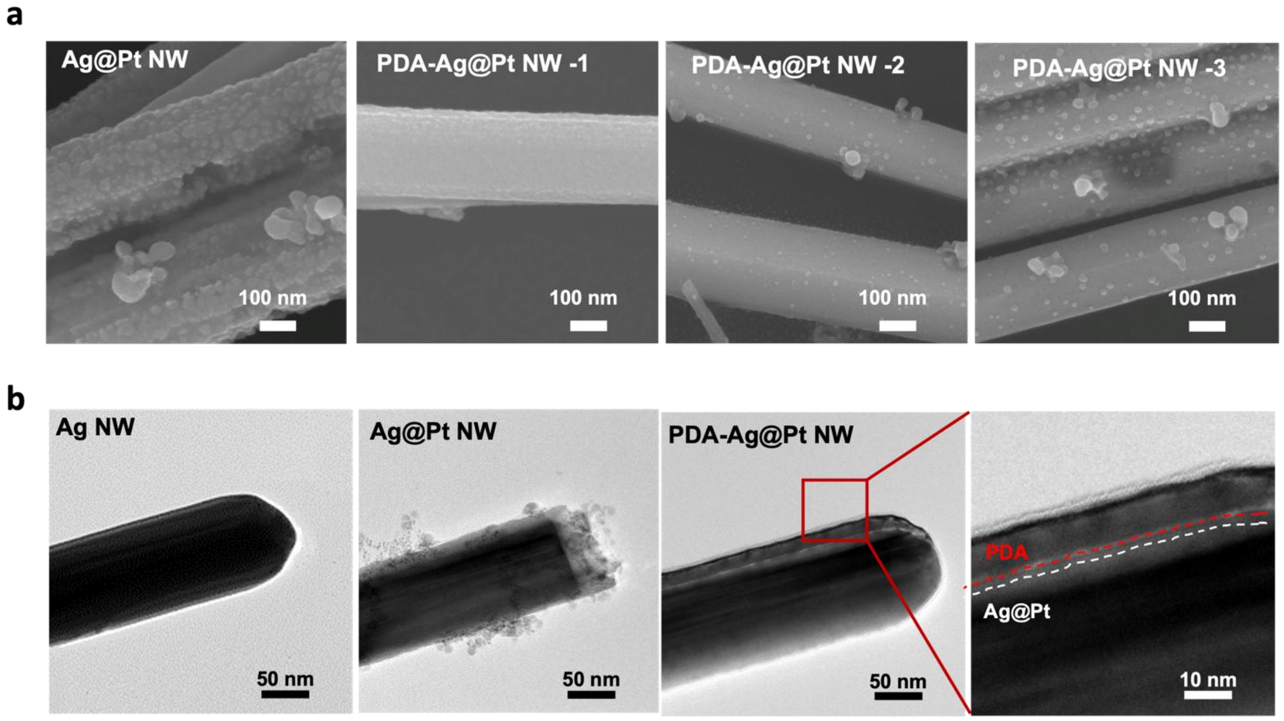

Figure S2 Characterization of AgNW structures. (a) SEM images showing the morphology of Ag@Pt NW and PDA-Ag@Pt NW samples at different stages of modification. (b) TEM images revealing the structural details of Ag NW, Ag@Pt NW, and PDA-Ag@Pt NW. The enlarged image provides a higher magnification view of the PDA-Ag@Pt NW surface, highlighting the Ag@Pt core-shell structure.

3. HRTEM and XPS analysis to identify the chemical state of Ag and Pt

The crystalline structures of Ag NW and Pt@Ag NW were analyzed using HRTEM, revealing distinct lattice fringes corresponding to different crystal planes.XPS analysis confirms the presence of C, O, N, Ag, and Pt in PDA-Pt@Ag NWs, with Ag, C, and O being predominant. The spectra indicate that Ag remains in its metallic state without significant oxidation. Similarly, Pt is present in its elemental form with minimal oxidation, suggesting the successful incorporation and stability of both metals within the PDA matrix.

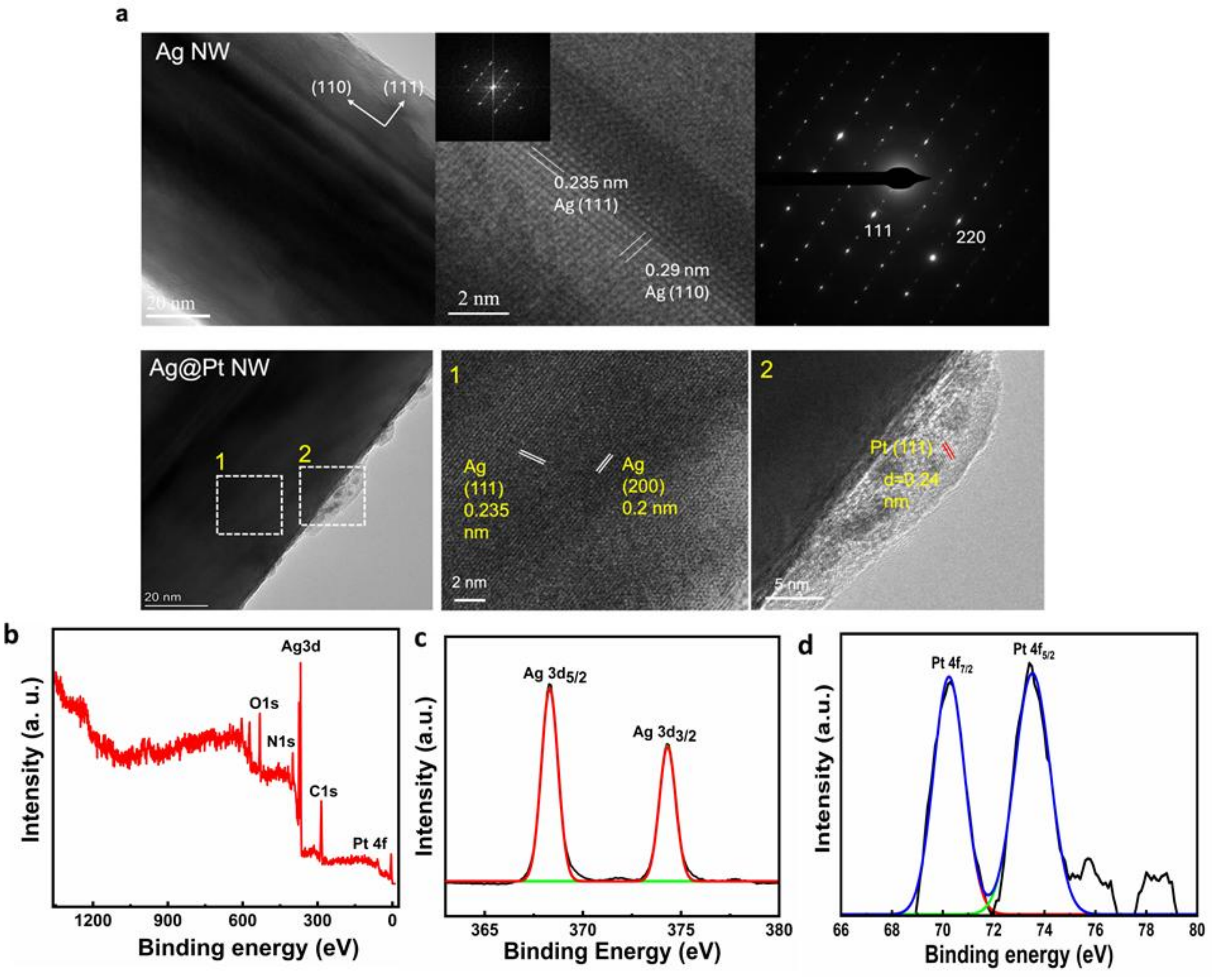

Figure S3 (a) HRTEM of Ag and Pt@Ag NWs. (b) Full scan XPS survey spectra of PDA-Pt@Ag NW, XPS spectra of (c) Ag 3d (d) Pt 4f.

4. Contact impedance measurement for wearable electrode application

Contact impedance was measured using two electrodes placed 2 cm apart on the arm. The PDA-PAM/NW electrode demonstrated lower impedance than the commercial Ag/AgCl electrode across all frequencies, indicating its effective skin-electrode interface.

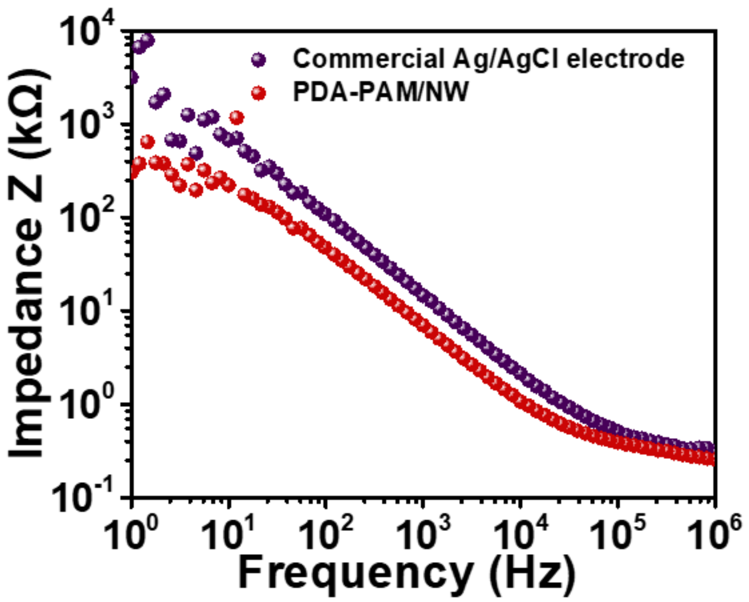

Figure S4 Bode plots showing the impedance values in a frequency window.

5. Enhanced Sensitivity of PDA-PAM/NW Electrodes for sEMG Detection

PDA-PAM/NW electrodes exhibit higher MUP amplitude due to better skin compliance, making them more sensitive to muscle contractions and vibrations.

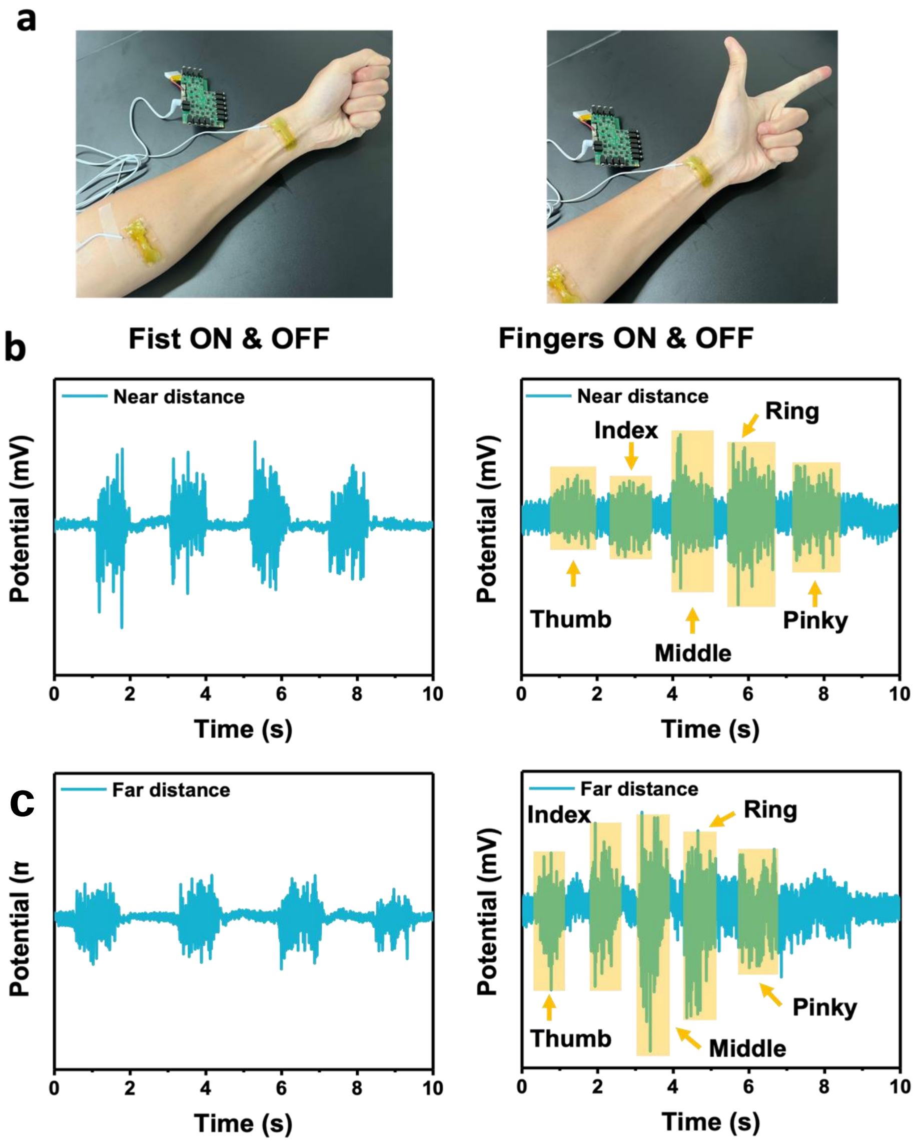

Figure S5 (a) Electromyography (EMG) measurements comparing fist and finger movements at varying distances. (b) Potential vs. time plots for fist ON & OFF movements at near and far distances, respectively. (c) Potential vs. time plots for individual finger movements (thumb, index, middle, ring, and pinky) at near and far distances.
